# Real-World Methotrexate Dose on Clinical Effectiveness and Structural Damage of Certolizumab Pegol With Rheumatoid Arthritis

**DOI:** 10.3389/fmed.2021.643459

**Published:** 2021-04-21

**Authors:** Yuji Nozaki, Toshihiko Hidaka, Jinhai Ri, Tetsu Itami, Daisuke Tomita, Akinori Okada, Chisato Ashida, Fusayo Ikeda, Atsuhiro Yamamoto, Keiko Funahashi, Koji Kinoshita, Tsukasa Matsubara, Masanori Funauchi, Itaru Matsumura

**Affiliations:** ^1^Department of Hematology and Rheumatology, Kindai University Faculty of Medicine, Osaka-Sayama, Japan; ^2^Institute of Rheumatology, Zenjinkai Shimin-No-Mori Hospital, Miyazaki, Japan; ^3^Matsubara Mayflower Hospital, Kato, Japan

**Keywords:** cytokines, rheumatoid arthritis, biological, X ray, DAS28-ESR, certolizumab pegol

## Abstract

**Objective:** Rheumatoid arthritis (RA) treatments have markedly advanced with the introduction of biological agents, e. g., tumor necrosis factor (TNF) inhibitors. TNF inhibitors are demonstrated to be quite effective in combination with methotrexate (MTX), and sufficient doses of both agents are important to control RA's disease activity. However, not all RA patients can be treated with high-dose MTX due to contraindications related to the antimetabolite action of MTX or to tolerability concerns. In daily practice, this has resulted in reduced effectiveness of TNF inhibitors. We sought to determine whether the concomitant use of dose of MTX affected the clinical effectiveness, retention rate, and side effects of certolizumab pegol (CZP) for treating RA in a real-world setting. CZP is a pegylated–conjugated Fab' fragment of a humanized anti-TNF antibody that has high affinity to TNF.

**Patients and Methods:** We divided Japanese RA patients treated with CZP (*n* = 95, 25–83 years old) into groups based on those with (*n* = 65) and without (*n* = 30) concomitant MTX and those treated with a high dose (≥8 mg, *n* = 41) or low dose (1– <8 mg, *n* = 24) of MTX. We retrospectively analyzed the concomitant MTX doses' effects and side effects and the patient retention rate.

**Results:** There were no significant differences among the CZP groups with and without MTX or the groups receiving the high vs. low MTX doses in the retention rate, the low disease activity rate, or the inhibitory effect in radiographic joint damage.

**Conclusion:** CZP has the potential to be a useful biological agent to control RA's disease activity and the bone destruction in patients who cannot tolerate a sufficient MTX dose.

## Introduction

The treatment of rheumatoid arthritis (RA) has greatly progressed with the use of new biological agents including tumor necrosis factor (TNF) inhibitors, which have been observed to be efficacious for RA when administered in combination with the chemotherapy agent methotrexate (MTX). The recommended dose of MTX in the USA and Europe is 15–25 mg/week (1–3); the maximum dose in Japan is 16 mg/week. High-dose MTX has been demonstrated to increase the effectiveness of TNF inhibitors in early-stage RA and established RA. For example, in the Combination Therapy with Adalimumab in Subjects with Early Rheumatoid Arthritis (CONCERTO) study, high-dose (i.e., 20 mg/week) MTX administered in a regimen with the TNF inhibitor adalimumab (ADA) resulted in a three-fold higher remission rate than low-dose (2.5 mg/week) MTX with ADA in bio-naive patients with early-stage RA (4). However, high-dose MTX cannot be used for all patients for reasons such as tolerability concerns, contraindications related to its antimetabolite action, and lack of effectiveness (4).

Certolizumab pegol (CZP) is used to treat RA. CZP is a pegylated–conjugated Fab′ fragment of a humanized anti-TNF antibody that has high affinity to TNF. CZP does not have an Fc region; its use may therefore avoid the risk of Fc-mediated effects such as complement-dependent cytotoxicity and antibody-dependent cellular cytotoxicity, which have been observed *in vitro*; attachment of the polyethylene glycol moiety of CZP to the Fab' fragment yields a molecule with a plasma half-life of ~2 weeks (5). Patients with early-stage RA have been treated with MTX in combination with CZP, and the effectiveness and safety of such a regimen were assessed in the Certolizumab-Optimal Prevention of joint damage for Early RA (C-OPERA) study (6). In the randomized, placebo-controlled J-RAPID trial, CZP with MTX (average 11.4 ± 3.1 mg/week) provided a rapid reduction in RA signs and symptoms and the inhibition of structural joint damage in Japanese patients with active RA who had an inadequate response to MTX (7).

However, there has been no investigation of the effects of the dose of MTX on the efficacy of CZP and bone destruction in patients with established RA. We conducted the present prospective study to determine whether a concomitant dose of MTX affected CZP's treatment of RA and the retention rate. We divided CZP-treated patients with RA into those treated or not treated with concomitant MTX. We also classified the patients who were treated with concomitant MTX into two groups based on their average weekly dose of MTX: the low-dose (LD) patients who received 1– <7 mg MTX and the high-dose (HD) patients who received ≥8 mg MTX. We prospectively examined the effects of these concomitant MTX dose by using registry data on actual clinical use.

## Patients and Methods

### Patients

We examined the cases of 95 Japanese individuals diagnosed with RA based on the American College of Rheumatology (ACR) criteria (8) who had had active RA for ≥6 months despite treatment with conventional synthetic disease-modifying antirheumatic drugs (csDMARD). We excluded patients who had any of the following: other connective tissue disease with joint symptoms; hepatitis B, hepatitis C, or human immunodeficiency virus; a serious or opportunistic infection within 6 months prior to study enrollment; active tuberculosis; or chronic infectious diseases.

The enrolled patients were 25–83 years old and received a stable MTX dose (up to 16 mg/week). They meet the following additional criteria: ≥6 or more swollen joint count (SJC) and ≥6 tender joint count (TJC) and ≥2 of the following: a C-reactive protein (CRP) value ≥2.0 mg/dl; an erythrocyte sedimentation rate (ESR) ≥28 mm/h; a global health score ≥20 mm on a 0–100 mm patient's visual analog scale (PtVAS) in which 0 is the best score and 100 is the worst; radiography-documented evidence of bone erosion; and positive status for rheumatoid factor (RF) or anticyclic citrullinated peptide antibodies (ACPA). All of the patients underwent screening for latent and active tuberculosis.

We divided the 95 RA patients treated with CZP into those concomitantly treated with (*n* = 65) vs. without (*n* = 30) MTX. We also divided the CZP + MTX-treated patients into those treated with low-dose (1– <8 mg) MTX (*n* = 24; the LD group) or high-dose (≥8 mg) MTX (*n* = 41, the HD group). Concomitant treatment with an oral corticosteroid was permitted, e.g., a stable dose ≤10 mg of prednisolone(PSL)/day or an equivalent.

The treatment regimens were as follows. For weeks 0, 2, and 4, the patients received CZP (200 or 400 mg) subcutaneously with or without a 400 mg CZP loading dose. The patients were then treated every other week with 200 or 400 mg CZP, with or without a concomitant high or low dose of MTX through the follow-up. For the patients being treated with PSL, decreased treatment effectiveness was observed with the dose.

### Compliance With Ethical Standards

The patients were enrolled from March 2009 to April 2020 and were treated at Matsubara Mayflower Hospital, Zenjinkai Shimin-no-mori Hospital, and Kindai Hospital in Japan. The study was conducted in accord with the principles of the Helsinki Declaration of 1983 and was approved by the Research Ethics Committee of Kindai University of Medicine (30–2688). For this prospective cohort study, the patients' fully informed consents were obtained with written agreement.

### Clinical Assessments

At baseline, the patients' demographic characteristics were obtained (e.g., sex, age, disease duration, and current therapy). At each control visit, the laboratory tests below were performed. Each patient's value of RF (U/ml), ACPA (U/ml), and matrix metalloproteinase-3 (MMP-3; ng/ml) were also measured at baseline.

The following data were obtained at each visit from baseline to 12 months: the patients' scores on the Disease Activity Score assessing 28 joints with the erythrocyte sedimentation rate (DAS28-ESR), their TJC and SJC among 28 joints (both assessed by the patient's treating physician), the PtVAS score, and two laboratory parameters, i.e., CRP (mg/dl) and ESR (mm/h). We used the established definitions for evaluating RA disease activity. Regarding the categories of DAS28-ESR disease activity (9), we divided the patients into those at remission (DAS28-ESR <2.6) and those not in remission: low, 2.6 ≤ DAS28-ESR <3.2; moderate, DAS28-ESR 3.2–5.1; or high disease activity, DAS28-ESR >5.2. We defined clinical remission as the achievement of a DAS28-ESR value <2.6 and ≤1 on all four of the following ACR/European League against Rheumatism (EULAR) Boolean-based criteria (10): the number of TJC and SJC, the CRP and the PtVAS (the 100-mm visual analog scale data were converted to centimeters).

As the endpoint for the patients' clinical response to the treatments, we used their DAS28-ESR scores at 1, 3, 6, and 12 months. We also evaluated the patients' EULAR responses (11) at 1, 3, 6, and 12 months. Each patient's physical function was evaluated at baseline based on the Health Assessment Questionnaire Disability Index (HAQDI) (12).

### Radiographic Assessment

At baseline and at 12 months, plain radiographs of the patient's hands and feet were obtained, evaluated, and scored using both the Steinbrocker class and the modified Sharp/van der Heijde scoring system (13, 14). Two readers who were blinded to the patients' sequence and treatment reviewed the baseline and 12 months radiographs, and previous radiographs were reread concurrently with year 2 films.

We also calculated the mean changes from the baseline in the erosion score (ES), the modified total Sharp score (mTSS), and the joint space narrowing (JSN) score with the possible range of 0–390. We defined radiographic non-progression as an mTSS score ≤0.5. The smallest detectable change, an estimate of the measurement error between readers of the films (15), was also used.

### Safety

Each patient's treating physician recorded any adverse events (AEs) and made necessary treatment adjustments in accord with the study protocol. Any adverse reaction resulting in any of the following outcomes was considered a serious AE: hospitalization or prolongation of hospitalization, a life-threatening condition or death, a significant or permanent disability, a malignancy, a congenital abnormality, or a birth defect. Chest radiographs, electrocardiograms, and laboratory values, i.e., hematological, blood chemistry, and urinalysis values, were also evaluated.

### Statistical Analyses

Continuous variables were examined with summary statistics of the mean and standard deviation (SD) or the median and interquartile range (IQR) when appropriate. The categorical variables are shown as percentages. We used the Mann–Whitney test to compare independent means and the χ^2^ test to evaluate the relationships between categorical variables. The survival (retention) rate of this CZP-treated cohort was assessed by obtaining the Kaplan–Meier curves, and we used the log-rank test to examine the differences in retention curves. When data were missing, they were imputed (with the use of linear extrapolation for mTSS data) and with the last observation carried forward (LOCF) for all other effectiveness variables. Continuous variables were assessed using analysis of variance (ANOVA). Tukey's *post-hoc* analyses were computed when significant F ratios were obtained. Statistical significance was defined as a *p* < 0.05. All of the statistical analyses were performed with the GraphPad Prism program (GraphPad Software, San Diego, CA, USA) or JMP software (SAS, Cary, NC).

## Results

### The Patients' Characteristics

We retrospectively analyzed the cases of 95 RA patients treated with CZP with (*n* = 65) or without concomitant MTX (*n* = 30). Of the patients treated with MTX, 41 were treated with high-dose MTX (the HD group), and the other 24 patients were treated with low-dose MTX (the LD group) continuously throughout the study period. These groups' baseline characteristics, laboratory findings, and treatment are summarized in Table 1.

**Table 1:** The 95 RA patients' baseline clinical and laboratory data, and treatment information.

	**CZP with MTX (LD)**	**CZP with MTX (HD)**	**CZP with MTX**	**CZP without MTX**	**All CZP**	**p**
No. of patients	24	41	65	30	95	
Age, years	53.0 ± 15.9	49.7 ± 15.2	50.7 ± 15.4	54.6 ± 16.8	51.7 ± 15.8	0.3
Female, (%)	87.0	68.3	75.4	79.3	73.0	0.3
Disease duration, months	69.0 [16.0–129.0]	60.0 [13.5–114.0]	66.0 [15.0–119.5]	54.0 [28.0–106.0]	62.0 [22.0–114.0]	0.6
Bio naïve, %	59.1	61.0	60.3	67.9	61.2	0.8
RF, positive (%)	76.2	66.7	70.4	73.1	70.7	0.8
RF titers, IU/ml [IQR]	51.0 [12.8–109.0]	30.0 [5.8–94.2]	40.5 [6.1–94.2]	61.5 [8.5–163.5]	45.5 [7.3–122.7]	0.6
ACPA, (%)	73.3	73.1	73.2	76.5	74.1	1.0
ACPA titers, Al/ml [IQR]	37.2 [1.4–72.9]	35.7 [1.7–143.7]	37.2 [1.7–128.9]	55.7 [0.3–122.0]	37.8 [1.4–120.0]	0.9
CRP, mg/dl [IQR]	0.7 [0.2–2.6]	1.0 [0.2–2.9]	1.0 [0.2–2.6]	1.3 [0.4–2.5]	1.0 [0.3–2.6]	0.9
ESR, mm/h [IQR]	34.9 ± 21.1	39.1 ± 29.1	37.6 ± 26.4	43.7 ± 31.1	39.5 ± 27.9	0.5
MMP-3, ng/ml [IQR]	107.5 [53.7–432.5]	126.1 [52.8–126.1]	121.2 [52.9–287.9]	156.1 [105.8–330.4]	130.9 [66.8–336.8]	0.7
Tender joints, range 0–28 [IQR]	7.0 [2.0–10.0]	6.0 [2.0–10.0]	6.0 [1.0–10.0]	6.5 [4.0–11.5]	6.0 [2.0–10.8]	0.9
Swollen joints, range 0–28 [IQR]	5.0 [2.0–12.0]	5.0 [2.0–10.0]	5.0 [2.0–10.8]	5.0 [2.0–10.8]	5.0 [2.0–10.8]	1.0
Pt VAS, 0–100 mm	50.0 [25.0–71.0]	50.0 [20.0–76.0]	50.0 [23.5–70.8]	62.5 [40.0–70.0]	51.2 ± 27.4	0.6
DAS28-ESR	5.0 ± 0.4	5.0 ± 0.3	5.2 ± 1.6	5.0 ± 1.8	5.1 ± 1.7	0.8
CDAI score	25.2 ± 16.8	24.0 ± 15.4	24.4 ± 15.8	25.1 ± 12.6	24.6 ± 14.9	0.9
Total sharp score 0–448 [IQR]	25.3 [10.6–53.3]	7.3 [0.0–30.6]	14.5 [2.0–42.3]	5.5 [1.0–46.8]	12.5 [1.8–42.3]	1.0
Joint narrowing score [IQR]	10.3 [0.5–33.4]	5.0 [0.0–19.4]	7.5 [0.0–23.8]	3.5 [0.6–9.3]	6.0 [0.4–21.2]	0.2
Erosive score [IQR]	7.8 [1.0–26.5]	5.3 [0.0–13.9]	7.3 [0.4–23.5]	2.5 [0.5–7.9]	4.0 [0.5–20.8]	0.2
Steinbrocker stage (I/II/III/IV)	(19.1/42.9/23.8/14.3)	(39.5/29.0/15.8/15.8)	(30.4/33.9/19.6/16.1)	(29.2/33.3/20.8/16.7)	(30.0/33.8/20.0/16.2)	0.8
Steinbrocker class (I/II/III/IV)	(31.3/50.0/18.8/0.0)	(40.9/54.5/5.0/0.0)	(45.8/45.8/6.8/1.7)	(35.7/50.0/14.3/0.0)	(42.6/47.1/9.2/1.1)	0.4
HAQDI, range 0–3	1.0 [0.2–1.6]	1.0 [0.4–1.5]	1.0 [0.4–1.6]	1.1 [0.6–2.0]	1.0 [0.6–1.8]	0.5
CZP loading, %	78.9	81.8	79.8	80.4	80.0	0.7
MTX, %, mg/week [IQR]	100, 6.0 [4.0–6.0]	100, 10.0 [8.0–10.0]	100, 8.0 [6.0–10.0]	0, 0	69.1, 6.0 [0.0–8.0]	<0.0001^*^
PSL, %, mg/day [IQR]	34.8, 0.0 [0.0–2.5]	51.2, 0.5 [0.0–4.3]	46.9, 0.0 [0.0–4.0]	58.6, 3.3 [0.0–7.1]^*^	50.5, 0.0 [0.0–5.0]	0.1

*Values are median [25th−75th centiles] or mean (SD), unless otherwise indicated*.

* *p < 0.0001, CZP without MTX vs. other groups, CZP with MTX (LD) vs. other groups, CZP with MTX (HD) vs. other groups*.

*ACPA, anticitrullinated peptide antibody; CDAI, clinical disease activity index; CRP, C-reactive protein; DAS, disease activity score; ESR, erythrocyte sedimentation rate; HAQDI, Health Assessment Questionnaire Disability Index; IQR, interquartile range; MMP-3, matrix metalloproteinase-3; MTX, methotrexate; PSL, prednisolone; Pt-VAS, patient visual analog scale; RF, rheumatoid factor*.

Our analyses revealed no significant between-group background differences in age, sex, disease duration, percentage of bio-naive patients, the proportions of Steinbrocker stages and classes, steroid dose, positive percentage or titers of RF and ACPA, the DAS28-ESR, CDAI, and HAQDI, or the total Sharp score at baseline.

### Retention Rate

**Figure 1:**
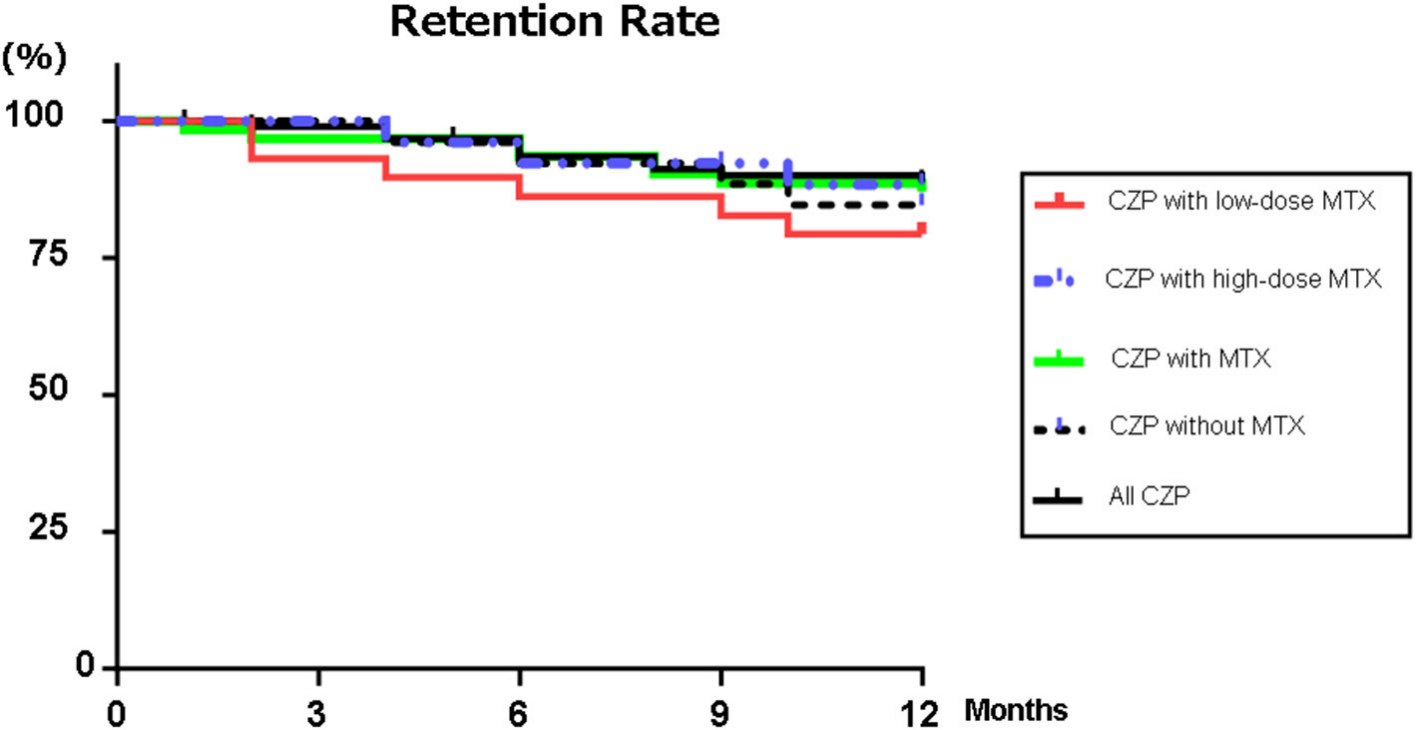
Retention rates. Kaplan–Meier curves for all CZP treatments, low- (2– <8 mg) and high-dose MTX (≥8 mg), with MTX, and without and with MTX group regarding the time of withdrawal due to lack of effectiveness, adverse effects, or the patient's request from the start of CZP to 12 months. CZP, certolizumab pegol; MTX, methotrexate.

Figure 1 illustrates the retention rates in the RA patients treated with CZP and with/without concomitant high- or low-dose MTX for the 12-month study period. Figure 1 also provides the overall retention rate over the study period, based on withdrawal from treatment for any of the following reasons: lack of effectiveness, one or more AEs, the patient's request, or sustained remission. At 12 months, the retention rates of all of the CZP treatments were 88.9% (Figure 1), and those in the without- and with-MTX groups were 84.6 and 87.1%, respectively. At 12 months, there was no significant difference between the CZP retention rates of the LD and HD groups at 79.3 and 88.3%, respectively.

### Clinical Effectiveness

The changes in the patients' baseline values demonstrated the disease activity at 1, 3, 6, and 12 months are summarized in Table 2: those of the DAS28-ESR and CDAI scores; the TJC28, SJC28, and PtVAS as clinical parameters; and the laboratory data of CRP, ESR, RF, and MMP-3. In all of the treatment groups, significant reductions were observed in the values of the DAS28-ESR, CDAI, TJC, SJC, and PtVAS as the clinical parameters and CRP as the laboratory data at 1, 3, 6, and 12 months compared to baseline. On the other hand, there were no significant differences in the MMP-3 value or RF titer at 1 month or during the 12-month treatment period compared to the baseline in any of the treatment groups.

**Table 2:** Clinical parameters and laboratory values during follow-up.

	**CZP+MTX (LD)**					**CZP+MTX (HD)**					**CZP with MTX**					**CZP without MTX**					**ALL CZP**					
	**Baseline**	**1 m**	**3 m**	**6 m**	**12 m**	**Baseline**	**1 m**	**3 m**	**6 m**	**12 m**	**Baseline**	**1 m**	**3 m**	**6 m**	**12 m**	**Baseline**	**1 m**	**3 m**	**6 m**	**12 m**	**Baseline**	**1 m**	**3 m**	**6 m**	**12 m**	***p*-value**
**Clinical parameters**																										
DAS28-ESR	5	4.1	3.6^**^	3.3^†^	2.7^#^	4.9	3.3^†^	2.8^#^	2.8^#^	2.4^#^	5	3.6^#^	3.0^#^	2.9^#^	2.5^#^	5.2	3.8^#^	3.3^#^	3.3^#^	3.1^#^	5	3.6^#^	3.1^#^	3.0^#^	2.7^#^	0.7
CDAI score	25.2	16.8	11.5^**^	9.7^†^	6.0^#^	23.5	10.0^#^	7.7^#^	6.4^#^	4.9^#^	23.9	12.1^#^	8.9^#^	7.6^#^	5.4^#^	25.4	12.8^#^	9.9^#^	8.6^#^	7.5^#^	24.4	12.3^#^	9.2^#^	7.9^#^	6.0^#^	0.8
Tender joints, range 0–28 [IQR]	8.4	5.8	4.3	3.4^*^	1.8^#^	7.4	3.5^#^	2.2^#^	2.3^#^	1.6^#^	7.7	4.1^#^	2.8^#^	2.7^#^	1.7^#^	7.9	3.8^#^	3.0^#^	2.5^#^	2.0^#^	7.7	4.0^#^	2.9^#^	2.6^#^	1.8^#^	0.8
Swollen joints, range 0–28 [IQR]	6.5	3.7	2.3^**^	1.4^†^	1.3^#^	6.6	2.5^#^	2.1^#^	2.3^#^	1.1^#^	6.5	2.9^#^	2.2^#^	2.0^#^	1.2^#^	6.9	3.8^#^	2.5^#^	1.6^#^	1.6^#^	6.6	3.1^#^	2.3^#^	1.9^#^	1.3^#^	0.9
Pt VAS, 0–100 mm	49.2	38.2	27.9^*^	25.1^*^	16.2^#^	49.2	21.6^†^	18.4^#^	17.4^#^	11.6^#^	49.2	27.2^#^	21.5^#^	20.1^#^	14.1^#^	55.8	29.7^#^	24.0^#^	24.9^#^	21.2^#^	51	27.9^#^	22.2^#^	21.5^#^	16.2^#^	0.6
**Laboratory parameters**																										
CRP, mg/dl [IQR]	1.7	0.7^*^	0.4^*^	0.3^**^	0.2^**^	2	0.6^**^	0.4^†^	0.6^**^	0.4^†^	2	0.6^#^	0.4^#^	0.5^#^	0.3^#^	1.9	0.8^#^	0.5^#^	0.7^#^	0.5^#^	1.9	0.7^#^	0.5^#^	0.5^#^	0.4^#^	0.8
ESR, mm/h [IQR]	34.9	24.6	21.8^*^	19.4^**^	19.3^*^	37.8	21.9^**^	18.5^†^	20.4^**^	17.5^†^	37.4	23.4^†^	20.0^#^	20.0^#^	18.1^#^	42.4	31.0^†^	27.7^#^	27.9^#^	28.1^#^	38.9	25.7^#^	22.0^#^	22.4^#^	21.1^#^	0.5
RF titer, IU/ml [IQR]	70.6	51.6	52.4	60.2	38	87.8	92.5	86.8	80.9	77.7	80.1	72.9	72.1	73.4	63.9	113.1	89	93.2	101.1	127.9	89.8	77.7	78.4	81.9	84.2	0.5
MMP-3, ng/ml [IQR]	214.8	235.8	101.2	119	97.5^*^	201.2	77.3	58.7	98.8^*^	83.4^**^	214.5	162	76.9^*^	105.6^**^	88.1^†^	227.3	111	119.6	122.0^*^	80.9^†^	218.2	146.9	94.7	110.4^#^	86.1^#^	0.6

*Serial changes in DAS28-ESR, CDAI, TJC, SJC, PtVAS as the clinical parameters and CRP, ESR, RF, and MMP-3 as the laboratory data from baseline in the patients with CZP in the combination of MTX who were classified into the following two groups according to the average weekly dose of concomitant MTX as LD group, 1 to <8 mg and HD group, 8 mg ≤. Statistical analysis was by a log-rank t-test. Values are median [25th−75th centiles] as tender joints, swollen joints, Pt VAS, CRP, RF titers, MMP-3, and the dosage of MTX and PSL or mean (SD) as DAS28-ESR, CDAI score, and ESR unless otherwise indicated. CDAI, Clinical Disease Activity Index; CRP, C-reactive protein; DAS, Disease Activity Score; ESR, erythrocyte sedimentation rate; MMP-3, matrix metalloproteinase-3; MTX, methotrexate; PSL, prednisolone; Pt-VAS, patient visual analog scale; RF, rheumatoid factor*.

* *p < 0.05*,

** *p < 0.005*,

† p < 0.001 and

# *p < 0.0001, baseline vs. the value at 1, 3, 6, and 12 months*.

*P-values at baseline were also analyzed and no significant differences in all treatment groups by ANOVA analysis*.

**Figure 2:**
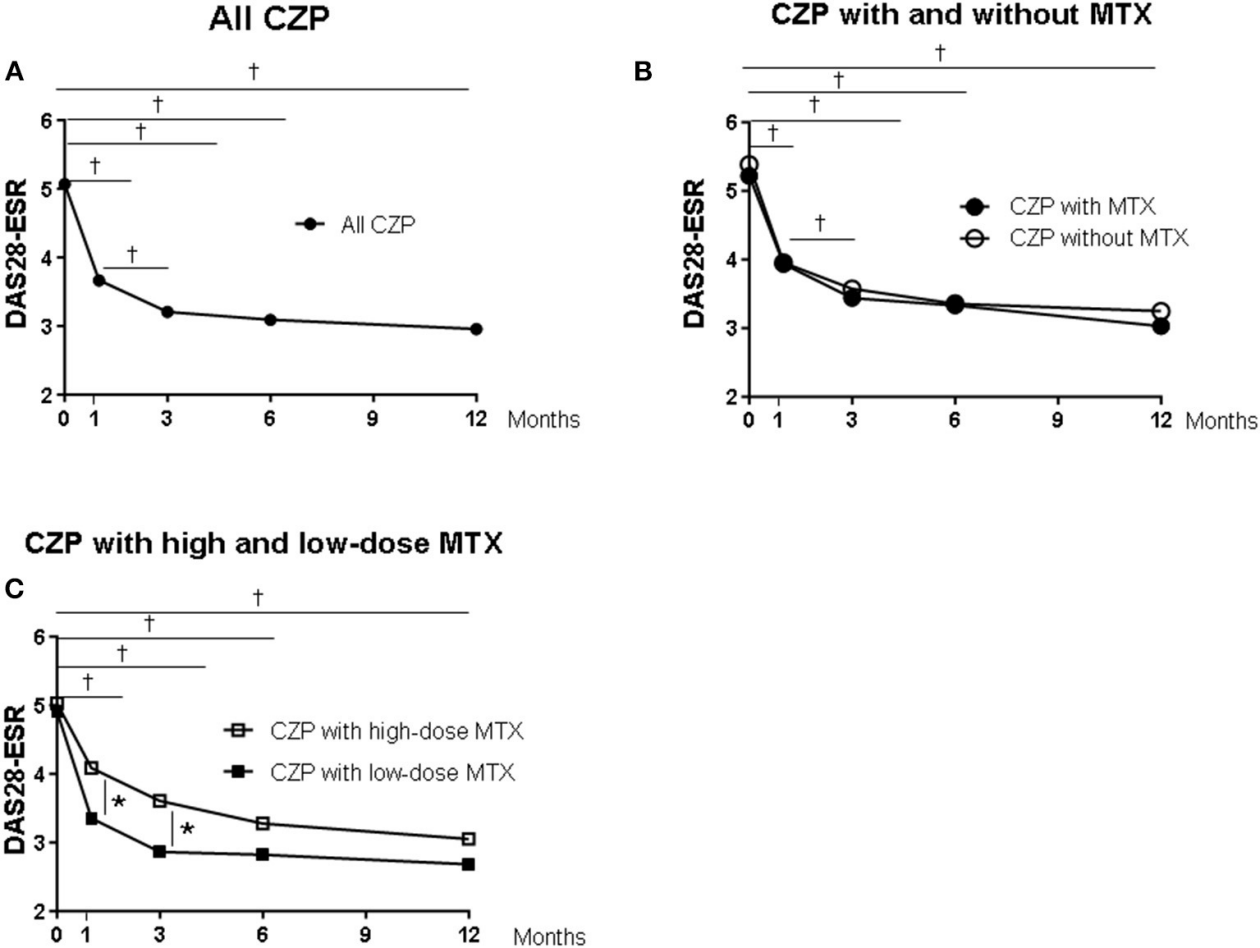
Clinical and laboratory data from baseline in certolizumab pegol (CZP) without and with methotrexate (MTX) in the clinical course. Serial changes in DAS28-ESR from baseline in **(A)** all CZP treatments, **(B)** with and without MTX, and **(C)** the low- and high-dose MTX. *p < 0.05 and ^†^*p* < 0.001, baseline vs. the value at 1, 3, 6, and 12 months. DAS28-ESR, Disease Activity Score assessing 28 joints with erythrocyte sedimentation rate.

Figure 2 provides the DAS-ESR values from baseline as the clinical response in the patients in each group. In the all-CZP-treatment cohort, the DAS28-ESR shows an early decline from baseline, and there were significant reductions at 1, 3, 6, and 12 months (Figures 2A–C). No significant difference in the DAS28-ESR was detected between the CZP without- and with-MTX groups over the 12 months (Figure 2B). However, the DAS28-ESR values were significantly lower in the group of patients treated with CZP and LD-MTX compared to those treated with HD-MTX at 1 and 3 months, although the difference between these groups disappeared at 6 and 12 months (Figure 2C).

**Figure 3:**
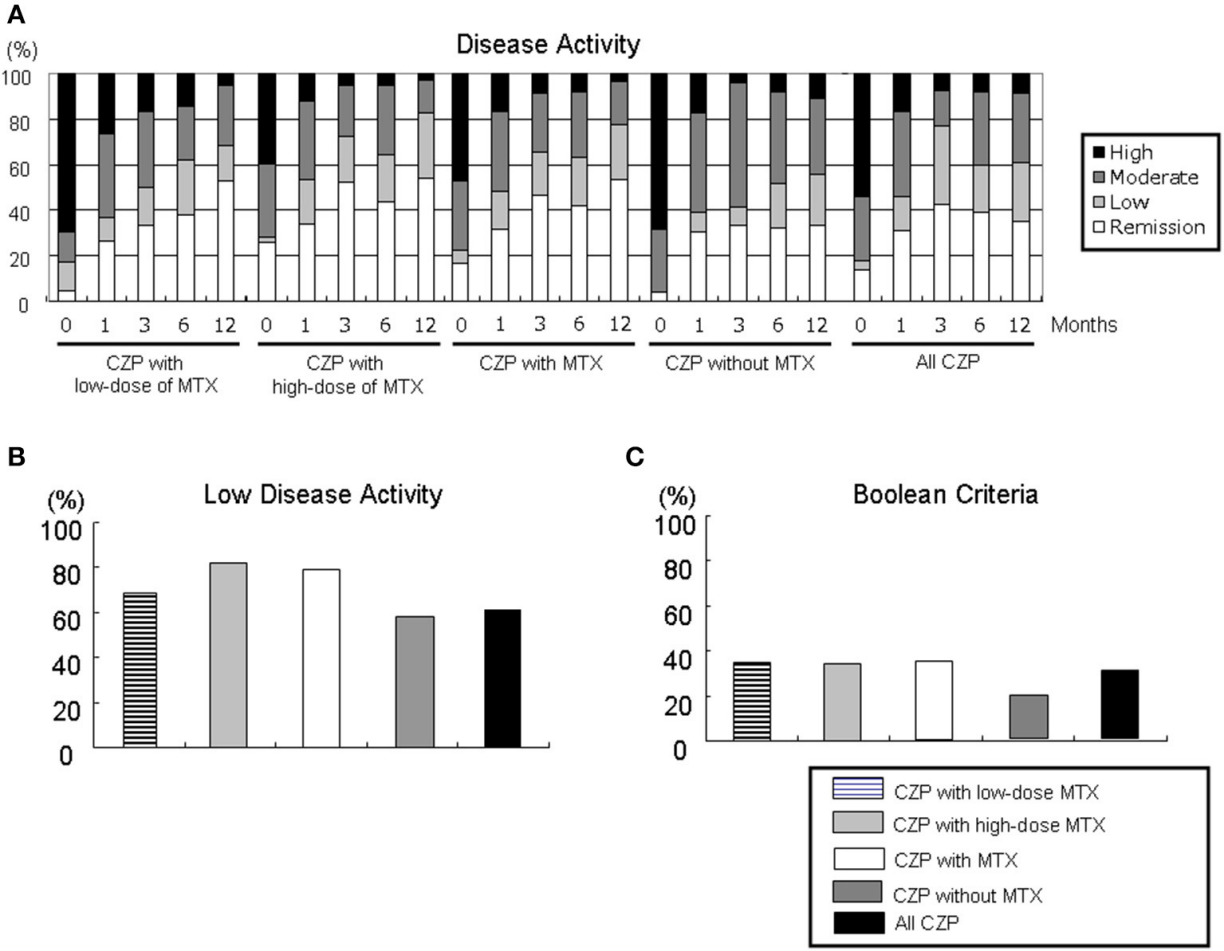
Disease activity in DAS28-ESR and ACR/EULAR Boolean-based criteria. By comparing the RA patients' DAS28-ESR scores at baseline and at 1, 3, 6, and 12 months, it is possible to define **(A)** disease activity, **(B)** low disease activity including remission, and **(C)** Boolean-based criteria in RA patients with the low- and high-dose MTX, with MTX, and without and with MTX, and all CZP treatments. The disease activity and criteria in DAS28-ESR were categorized as follows. DAS28 criteria: ■ high: 5.1 <DAS28; 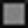 moderate, 3.2 ≤ DAS28 ≤ 5.1; 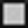 low, 2.6 ≤ DAS28 <3.2; □ remission: DAS28 < 2.6. ACR/EULAR Boolean-based remission: TJC, SJC, PtVAS, and CRP all ≤1. CRP, C-reactive protein; EULAR, European League Against Rheumatism; PtVAS, patient's visual analog scale; RA, rheumatoid arthritis; SJC, swelling joint count; TJC, tender joint count.

We evaluated the proportions of disease activity by assessing the DAS28-ESR in the all-CZP cohort, the LD and HD-MTX groups, and the groups with and without MTX (Figure 3A). At 12 months, the proportions of patients with low disease activity (LDA) including remission based on DAS28-ESR criteria were as follows: LD-MTX, 68.4%; HD-MTX, 82.9%; with MTX, 77.8%; without MTX, 55.5%; and all CZP treatments, 60.9% (Figure 3B). We also evaluated remission based on ACR/EULAR Boolean-based criteria at 12 months, and we observed no significant differences: LD-MTX, 35.0%; HD-MTX, 34.3%; with MTX, 34.6%; without MTX, 19.1%; and all CZP treatments, 30.3% (Figure 3C).

**Figure 4:**
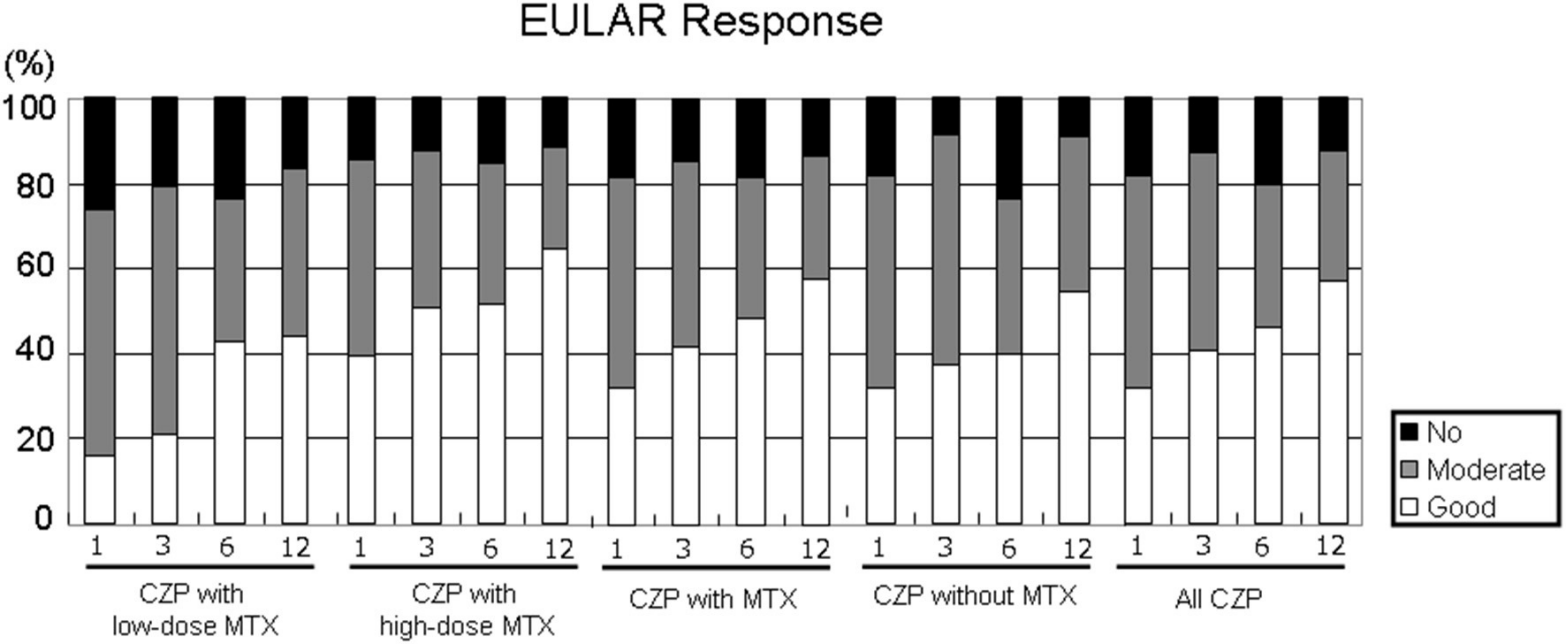
Disease activity in EULAR response. By comparing the RA patients' DAS28-ESR scores at baseline and at 1, 3, 6, and 12 months, it is possible to define the EULAR response in RA patients in the low- and high-dose MTX, with MTX, and without and with MTX, and all CZP treatments. The EULAR responses were categorized as follows. ■ no response: DAS28 improvement ≤0.6 and DAS28 improvement >0.6 and ≤1.2 in present DAS28 >5.1. 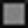 Moderate response: DAS28 improvement >0.6 and ≤1.2 in present DAS28 ≤3.2, and >3.2 and ≤5.1; DAS28 improvement >1.2 in present DAS28 >5.1, and >3.2 and ≤5.1. □ Good response: DAS28 improvement >1.2 in present DAS28 ≤3.2.

Figure 4 shows the EULAR responses at 1, 3, 6, and 12 months in all five groups. The rate of responders (good or moderate response) at 12 months was as follows: LD-MTX, 83.3%; HD-MTX, 88.2%; with MTX, 86.6%; without MTX, 91.0%; and all CZP treatments, 87.9%. At 1 and 3 months, the rate of good responders tended to be lower in the LD group compared to the HD group. The proportion of non-responder patients at 12 months were as follows: LD-MTX, 16.4%; HD-MTX, 15.4%; with MTX, 13.5%; without MTX, 9.1%; and all CZP treatments, 12.0%. There were no significant differences among any of the groups in the rate of responders or the rate of non-responders in EULAR response during the study period.

### Radiographic Progression

**Figure 5:**
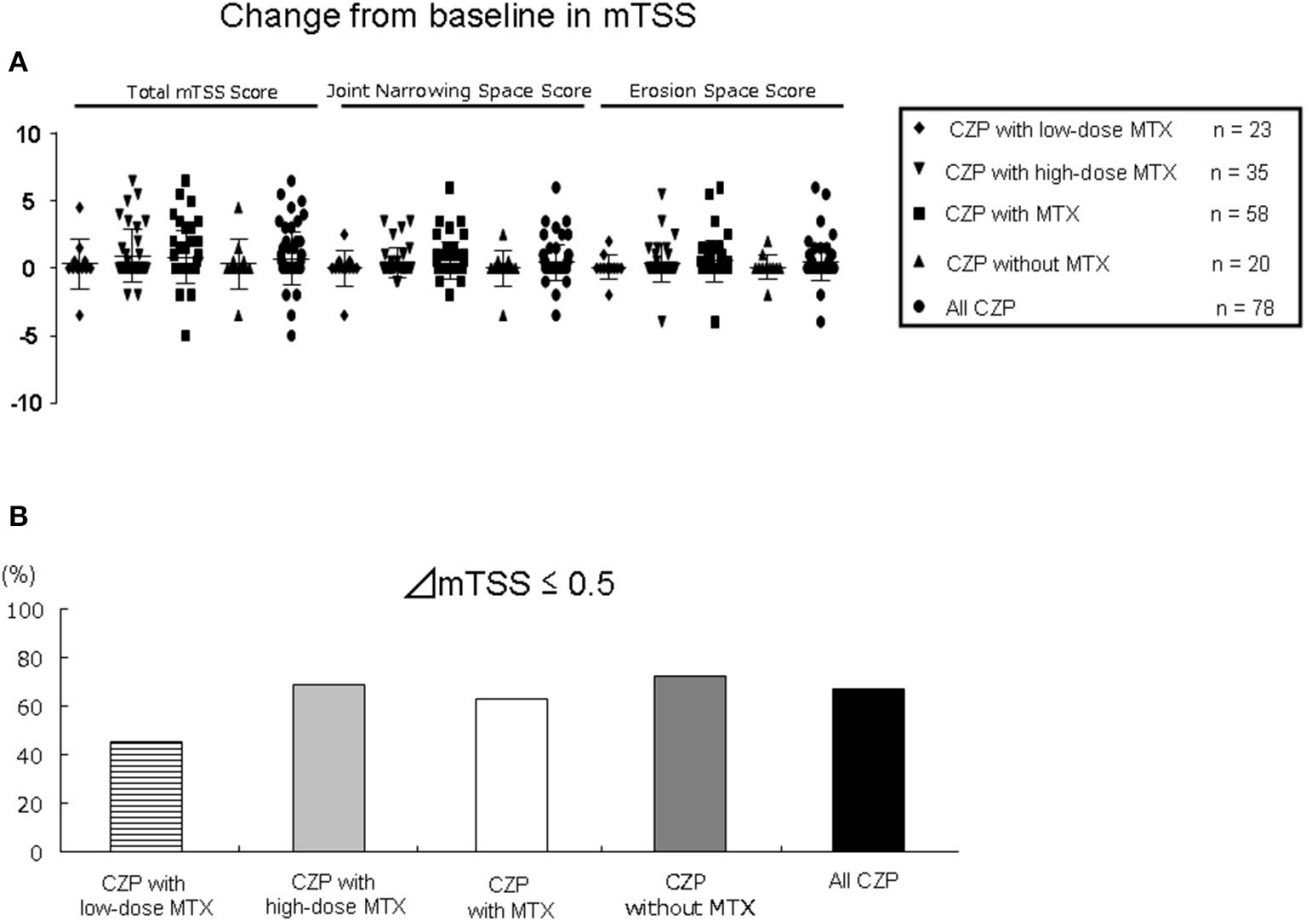
Joint damage in radiographic assessment. The progression of joint damage in RA patients in the low- and high-dose MTX, with MTX, and without and with MTX, and all CZP treatments according to the modified total Sharp score (mTSS) at 12 months. **(A)** The change in mTSS from baseline. **(B)** The rate of patients with progression, no change, or improvement in the mTSS (ΔmTSS ≤0.5).

Seventy-eight radiographs were assessed in the 95 patients treated with concomitant MTX (LD, *n* = 23 and HD, *n* = 35) and in 20 patients without MTX at 12 months (Figure 5). Regarding the joint space narrowing (JSN) score, erosion number, and TSS at baseline, all of the groups had similar values (Table 1). As shown in Figure 5A, at 12 months, there were no significant differences in all-CZP treatment cohort, LD and HD-MTX groups, and groups with and without MTX in the mean changes in JSN at 0.4 (0.1–0.8), 0.0 (−0.8–0.8), 0.4 (0.1–0.8), 0.5 (0.1–0.9), and 0.0 (−0.8–0.8); or in erosions at 0.4 (0.1–0.8), 0.1 (−0.5–0.7), 0.4 (−0.1–0.9), 0.5 (0.1–1.0), and 0.1 (−0.5–0.7); or in the TSS at 0.7 (0.2–1.3), 0.3 (−0.9–1.6), 0.9 (0.3–1.6), 0.8 (0.2–1.4), and 0.3 (−0.9–1.6), respectively. The percentages of patients with no progression of joint damage (i.e., improved or no change; ΔmTSS ≤0.5) were as follows: LD-MTX, 45.5%; HD-MTX, 68.6%, with MTX, 63.0%; without MTX, 72.2%; and all CZP treatments, 67.2% (Figure 5B).

### Adverse Events

Table 3 lists the treatment AEs: pneumonia, herpes zoster, and interstitial lung disease as serious AEs, and upper respiratory infection, thrombocytopenia, and rash during the 12 months after the start of CZP administration. During the 12-month study period, there was no significant difference in the cumulative rate of any of the AEs among any of the patient groups: LD-MTX, 17.1%; HD-MTX, 25.0%; with MTX, 21.5%; without MTX, 23.3%; and all CZP treatments, 21.1%. Serious infections occurred in 7.7% of the CZP with MTX group [pneumonia (*n* = 3), interstitial lung disease (*n* = 1)] and in 10.0% of the CZP without MTX group (one patient with pneumonia and herpes zoster). There were no significant differences in the rate of serious AEs between the HD and LD groups (7.3 and 4.2%). However, the incidence of any AE tended to be lower incidences in the LD group than the HD group. None of the patients treated with CZP experienced any AE that was serious enough to result in treatment discontinuation.

**Table 3:** Adverse events observed over 12 months resulting in CZP administration.

**Treatment group**	**CZP+MTX (LD)**	**CZP+MTX (HD)**	**CZP +MTX**	**CZP without MTX**	**All CZP**	***p***
**No. of patients**	**24**	**41**	**65**	**30**	**95**	
Any AEs, *n* (%)	7 (17.1)	6 (25.0)	14 (21.5)	7 (23.3)	20 (21.1)	0.9
Upper respiratory infection, *n* (%)	5 (12.2)	3 (12.5)	8 (12.3)	3 (10.0)	10 (10.5)	0.7
Thrombocytopenia, *n* (%)	0 (0.0)	1 (2.4)	1 (1.5)	0 (0.0)	1 (1.1)	1.0
Rash, *n* (%)	0 (0.0)	0 (0.0)	0 (0.0)	1 (3.3)	1 (1.1)	0.8
Serious AE, *n* (%)	1 (4.2)	3 (7.3)	5 (7.7)	3 (10.0)	7 (7.4)	0.6
Pneumonia, *n* (%)	1 (4.2)	2 (4.9)	3 (4.6)	1 (3.3)	4 (4.2)	0.9
Herpes zoster, *n* (%)	0 (0.0)	0 (0.0)	0 (0.0)	1 (4.5)	1 (1.1)	0.5
Interstitial lung disease, *n* (%)	0 (0.0)	1 (2.4)	1 (1.5)	0 (0.0)	1 (1.1)	0.3
Death, *n* (%)	0 (0.0)	0 (0.0)	0 (0.0)	0 (0.0)	0 (0.0)	1.00

*AE, adverse event; CZP, certolizumab pegol; MTX, methotrexate*.

## Discussion

In this study, there were no differences in clinical effectiveness and radiographic inhibition in patients treated with CZP, with the high or low MTX doses or without concomitant MTX in Japanese patients with RA. MTX has very good cost effectiveness and a good efficacy/toxicity ratio, but its toxicity remains a concern. The potential AEs associated with MTX (e.g., nausea, headache, fatigue, mucositis, and alopecia) have been examined in part because they are a major cause of drug withdrawal (16–18). MTX-associated hepatotoxicity is frequent in RA patients, but mild and transient, not needing either drug interruption or dose adjustment in the majority of these cases (19). The exact mechanism of MTX toxicity is not yet clear. The same or differing metabolic pathways may be involved in the mechanisms underlying the efficacy and toxicity of MTX.

The use of a TNF antagonist has been advised when active disease remains after the optimization of non-biological therapy with DMARDs. For RA patients with high disease activity and risk factors associated with poor prognosis (e.g., high ACPA or RF titer, erosive disease), the early initiation of biological therapy to reduce joint damage and functional decline is recommended (20). Combination therapy with a TNF inhibitor and MTX has been demonstrated to significantly improve the clinical manifestations of RA, toward the goal of achieving remission or low disease activity (1, 2, 20, 21).

The CONCERTO study of the TNF inhibitor adalimumab in early RA revealed a trend toward improved efficacy when the dose of MTX was increased from 2.5 mg/week to 20 mg/week (4). Conversely, in MTX-naive patients with early RA and one or more poor-prognosis factors, CZP + MTX was shown to significantly reduce structural damage and decrease disease activity, although there was no clear association between the MTX dose and joint suppression (22). The HIKARI study demonstrated significant clinical benefit, functional improvement, and structural protection in Japanese patients with RA treated with the TNF inhibitor etanercept without concomitant MTX (23).

Our present study is the first to confirm that the treatment of RA in a Japanese population with CZP without MTX (either as CZP monotherapy or in combination with a non-MTX DMARD) was effective in controlling their clinical signs/symptoms and reducing the radiographic progression of joint damage due to RA, and our results confirm the safety of CZP.

One of the reported effects of the concomitant use of MTX is a reduction in the antidrug antibody (ADA) formation. Krieckaert et al. demonstrated that the rate of appearance of antiadalimumab (AAA) as an ADA was ~30% with concomitant MTX at ≤10 mg, but the rate of AAA appearance was <20% at concomitant MTX doses ≥12.5 mg (24). In their investigation of Japanese patients, Miyasaka observed that the rate of AAA positivity was high at 44%, indicating that there is a higher rate of AAA formation in Japanese RA patients (25).

Certolizumab pegol is the only PEGylated TNF inhibitor. Polyethylene glycol (PEG) is a polyetherized repeating subunit of ethylene glycol of various molecular weights (5). PEG is commonly attached to proteins to increase their half-life *in vivo*. Certolizumab pegol is also a TNF inhibitor in which PEGs may optimize the delivery of neutralizing moieties by specifically targeting inflamed tissues in RA patients. In the inflamed synovium in RA, the disrupted endothelial tissue does not have the same barriers to diffusion as normal tissues and, therefore, may not be as effective as normal tissues (26).

Anti-CZP antibodies have been detected in 5–6.4% of RA patients treated with CZP monotherapy or with concomitant MTX (27). In the J-RAPID study, anti-CZP antibodies were observed in 1.2% of the 200- and 400-mg CZP groups, although the MTX dose was set at 6–8 mg/week in accord with the approved dose in Japan at the time of this clinical trial (7). The lower incidence of ADA among RA patients treated with CZP might have affected the retention rate, clinical effectiveness, and bone destruction.

The results of the RA (RAPID)2 randomized controlled trial demonstrated that a loading dose of 400 mg at weeks 0, 2, and 4, then CZP (200 or 400 mg) every other week in combination with MTX provided significant and rapid improvements in the signs and symptoms of RA after 24 weeks of treatment (28). We attempted to perform a subanalysis to determine the effectiveness of loading treatment, but the number of patients was not sufficient for a statistical analysis of loading vs. non-loading as the initial treatment between groups receiving a low or high dose of concomitant MTX. It is difficult to treat some RA patients because of the above-mentioned problems presented by MTX, and it can be difficult to increase the MTX dose; these difficulties contribute to a reduced retention rate and effectiveness (29–31).

We observed no significant differences among any of the five present patient groups in the rates of retention and clinical remission, in structural protection (by analyses of the mTSS with the scores of erosion and joint space narrowing), or the non-progression rate. The reduction in DAS28-ESR was significantly greater in the CZP with high-dose MTX group compared to the CZP with low-dose group at 1 and 3 months, but there was no significant difference at 6 months. The rate of low disease activity (remission and good responders) tended to be higher in the patients treated with CZP and high-dose MTX compared to those treated with low-dose MTX. Conversely, there was no significant difference in the Boolean-based criteria between the CZP with HD vs. LD groups. In the patients treated without MTX, the rates of achieving LDA and the Boolean-based criteria tended to be lower than those of the other groups. These findings suggested that rheumatologists and patients should both take part in decisions about treatment goals based on the disease activity, progression of structural damage, and the safety issues when considering a CZP-only regimen or a combination with MTX (low or high dose) in light of potential complications such as interstitial pneumonia, liver and renal dysfunction, gastrointestinal disorder, and blood disorder.

This study has some limitations to address. The lack of randomization and blinding may have resulted in bias due to the selection of the patients and their indications. In clinical practice, only patients with RA who fail to respond to MTX would receive CZP with MTX, and thus, our CZP with MTX groups are likely to have included more MTX-resistant patients than the CZP without MTX group. This may have affected the therapeutic effectiveness. Second, only Japanese RA patients were analyzed in this clinical study. In addition, the mean MTX dose (8 mg) in our cohort is low compared to similar RA studies from the European Union and USA (15–17 mg/week) (1–3). However, the mean MTX dose was 7.4 mg/week in phase II/III clinical trials conducted in Japan (7), and the dose used herein is a standard dose in Japan considering body weight and MTX metabolism (32).

The study population was rather small (*n* = 95), which may have resulted in low statistical power and low precision of the analysis. Gender, disease duration, RF and ACPA positivity, and disease activity at baseline may also be variables that influence efficacy and retention, and our adjustment for confounders may be inadequate due to the small sample size. Because this was a retrospective study, there were no predetermined treatment discontinuation criteria. Discontinuation of treatment at the discretion of each physician may also lead to bias. A randomized controlled trial is essential to establish the effectiveness of CZP treatment with or without MTX. Nevertheless, the results of our analyses demonstrated that CZP treatment with and without MTX were effective to reduce the disease activity of RA and the progression of bone destruction.

To date, there have been no reports on the effectiveness of CZP treatment with or without MTX or on the dose of MTX used to treat RA patients in a real-world setting, and our present investigation is the first clinical study. In all of the relevant treatment guidelines, MTX is described as effective, well-tolerated by patients, affordable, and recommended. However, high-dose MTX cannot be administered to all patients due to contraindications related to its antimetabolite action, tolerability concerns, or lack of effectiveness.

In this study, the rates of retention and clinical remission and the inhibitory effect on radiographic joint damage were not significantly different among the all-CZP-treatment group, the with-MTX group, the without-MTX group, and the high- and low-dose MTX groups. As real-world data in the era of biological agents in patients with RA, despite the limitations of a retrospective, non-blinded, and non-randomized study, and limited to a selected Japanese population, there were no differences in clinical effectiveness and radiographic inhibition in patients treated with CZP, with the high or low MTX doses, or without concomitant MTX. However, the present study has a short follow-up period as well as administered low-dose MTX. Since its selected population is Japanese, these results might be only valid in a population especially sensitive to higher doses of MTX such as the Asian RA patients. Further studies are needed, and the clinical effectiveness and radiographic assessment should be evaluated in prospective, randomized, large cohort, and longer term in the global trials. In summary, CZP shows the potential to be a useful biological agent to control the disease activity and bone destruction in patients with RA who cannot tolerate a sufficient dose of MTX.

## Data Availability Statement

The raw data supporting the conclusions of this article will be made available by the authors, without undue reservation.

## Ethics Statement

The studies involving human participants were reviewed and approved by the Research Ethics Committee of Kindai University of Medicine (30–2688). The patients/participants provided their written informed consent to participate in this study. Written informed consent was obtained from the individual(s) for the publication of any potentially identifiable images or data included in this article.

## Author Contributions

YN designed, performed, analyzed the study, drafted the manuscript, and analyzed the data. JR, DT, TI, AO, CA, FI, and AY are evaluated and scored using Steinbrocker class and the modified Sharp/van der Heijde scoring system. TH, TM, KF, KK, MF, and IM edited the manuscript. All authors contributed to the manuscript's revision, read, and approved the submitted version or confirms being the sole contributor of this work and has approved it for publication.

## Conflict of Interest

YN has received honoraria or research grant from AbbVie GK, Astellas Pharma, Asahi Kasei, AYUMI Pharmaceutical, Chugai Pharmaceutical Co., Eisai Co., Daiichi-Sankyo, MSD, Mitsubishi Tanabe Pharma Corp., Takeda, Ono, Otsuka Co., Pfizer, Janssen, and UCB Japan. The remaining authors declare that the research was conducted in the absence of any commercial or financial relationships that could be construed as a potential conflict of interest.
